# Autophagy maintains ubiquitination-proteasomal degradation of Sirt3 to limit oxidative stress in K562 leukemia cells

**DOI:** 10.18632/oncotarget.9592

**Published:** 2016-05-25

**Authors:** Yixuan Fang, Jian Wang, Li Xu, Yan Cao, Fei Xu, Lili Yan, Meilan Nie, Na Yuan, Suping Zhang, Ruijin Zhao, Hongbin Wang, Mengyin Wu, Xiaoying Zhang, Jianrong Wang

**Affiliations:** ^1^ Hematology Center of Cyrus Tang Medical Institute, Jiangsu Institute of Hematology, Collaborative Innovation Center of Hematology, Jiangsu Key Laboratory for Stem Cell Research, Soochow University School of Medicine, Suzhou, China

**Keywords:** Sirt3, Autophagy, ubiquitination-proteasome pathway, oxidative stress, erythroleukemia cells

## Abstract

Sirtuin protein family member 3 (Sirt3) has been suggested as a positive regulator in alleviating oxidative stress by acting on the mitochondrial antioxidant machinery in solid tumors; however, its role and regulation in hematological malignancies has been poorly understood. Here, we show that contrary to what has been reported in solid tumors, in K562 leukemia cells elevated Sirt3 was associated with mitochondrial stress, and depletion of Sirt3 decreased reactive oxygen species (ROS) generation and lipid oxidation, but increased the ratio of reduced glutathione (GSH) to oxidized glutathione (GSSG), suggesting an opposite role of Sirt3 in regulating oxidative stress in the leukemia cells. Notably, loss of autophagy by deletion of autophagy essential gene or by pharmacological inhibition on autophagic degradation caused a significant accumulation of Sirt3. However, induced activation of autophagy did not cause autophagic degradation of Sirt3. Furthermore, inhibiting proteasome activity accumulated Sirt3 in autophagy-intact but not autophagy-defective cells, and disrupting functional autophagy either genetically or pharmacologically caused significantly less ubiquitination of Sirt3. Therefore, our data suggest that basal but not enhanced autophagy activity maintains ubiquitination-proteasomal degradation of Sirt3 to limit lipid oxidative stress, representing an adaptive mechanism by which autophagy, in collaboration with the ubiquitination-proteasomal system, controls oxidative stress by controlling the levels of certain proteins in K562 leukemia cells.

## INTRODUCTION

Progressively elevated oxidative stress caused by ROS leads to DNA damage and subsequently malignant transformation. Moreover, extremely high levels of oxidative stress can cause irreversible damage to DNA, lipids and proteins, leading to gene mutations or macromolecular misfolding, aggregation, mitochondrial stress, and ultimately cell death. Therefore, mechanisms to overcome oxidative stress are needed in both normal cells and malignant cells. In the extreme oxidative stress conditions, mitochondrial autophagy, known as mitophagy, is an effective mechanism to remove mitochondrial stress in a living cell [1]. We have recently found that erythroleukemia cells acquire an alternative mitophagy capacity and this mitophagy remains functional even when canonical autophagy is defective, thereby enabling the leukemia cells to be more advantageous over normal hematopoietic cells in counteracting intracellular and extracellular extreme stresses [2]. However, whether autophagy functions directly in relieving oxidative stress in the leukemia cells remains an open question.

Sirtuin family of proteins consists of seven members and is implicated in an extensive array of cell function [3]. The Sirtuin protein member Sirt3 was initially identified as an NAD^+^-dependent protein deacetylase that regulates epigenetic modification of targeted proteins [4, 5]. Sirt3 regulates ROS through regulating the activity of magnesium superoxide dismutase which detoxifies superoxide to hydrogen peroxide [6–11], and promoting the expression of MnSOD and catalase, both of which are the targets of FOXO3A [12]. The Sirt3-dependent deacetylation of FOXO3A promotes its nuclear translocation and subsequently enhances its transcriptional activity [13, 14]. Sirt3 regulates mitochondrial fatty-acid oxidation by reversible enzyme deacetylation [15]. The unfolded protein response (UPR) has been reported in overcoming mitochondrial stress [16–18]. Reduction in Sirt3 levels results in elevated ROS levels, which is directly linked to the switch to glycolysis in solid tumor cells [10]. Sirt3 was further found to play a role in the UPR cascade of breast cancer cells, modulating the antioxidant machinery. Inhibition of Sirt3 in breast cancer cells undergoing proteotoxic stress severely impairs the mitochondrial network and results in cellular death [19]. Therefore, previous studies in solid tumors indicate that reduction of Sirt3 levels leads to an elevation in ROS levels by compromising the mitochondrial antioxidant machinery and Sirt3 functions in alleviating oxidative stress.

In this study, we report that contrary to what has been known in solid tumors, in K562 human leukemia cells, Sirt3 plays an opposite role in relieving oxidative stress. Basal but not enhanced autophagy activity maintains ubiquitination-proteasomal degradation of Sirt3 to limit ROS level, representing an adaptive mechanism by which autophagy, in collaboration with ubiquitination-proteasomal system, buffers oxidative stress in the leukemia cells.

## RESULTS AND DISCUSSION

### Sirt3 contributes to oxidative stress in K562 leukemia cells

**Figure 1:**
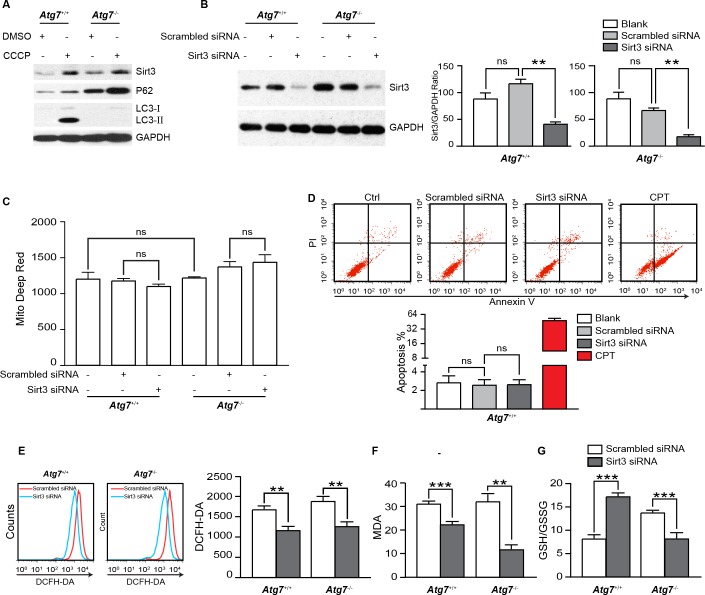
Examination of the role of Sirt3 in oxidative stress in K562 leukemia cells **A.** Immunoblotting analysis of Sirt3, P62 and LC3 proteins from mitophagy inducer CCCP treated *Atg7*^+/+^ and *Atg7*^−/−^ K562 cells. The cells were treated with 20 μM CCCP overnight. GAPDH served as a loading control. **B.** Knockdown of Sirt3 in *Atg7*^+/+^ and *Atg7*^−/−^ K562 cells. Cells were transfected with small inhibitory RNA targeting *Sirt*3 gene. The effects of siRNA on the expression of Sirt3 were evaluated by western blotting at 72 h after transfection. GAPDH served as a loading control. **C.** Flow cytometric analysis of mitochondrial mass in the Sirt3-depleted *Atg7*^+/+^ and *Atg7*^−/−^ K562 cells with Mitotracker Deep Red dye. **D.** Flow cytometric analysis of apoptosis of the Sirt3-depleted *Atg7*^+/+^ K562 cells. Cells were stained using FITC Annexin V apoptosis detection kit. Cells were treated with 5 μM Camptothecin for 3 h as a positive control. Upper panel: representative original flow data, and lower panel: statistic results. **E.** Flow cytometric analysis of ROS level in the Sirt3-depleted *Atg7*^+/+^ and *Atg7*^−/−^ K562 cells. Cells were stained at 10 μM DCF-DA for 15 min at 37°C. **F.** Determination of lipid perxodisation using MDA assay in the Sirt3-depleted *Atg7*^+/+^ and *Atg7*^−/−^ K562 cells. Cells were treated using a lipid peroxidation MDA assay kit, then detected at 532 nm on SpectraMax M5/M5e. Left panel: representative flow measurement data, and right panel: statistic results. **G.** Analysis on the ratio of GSH to GSSG in the Sirt3-depleted *Atg7*^+/+^ and *Atg7*^−/−^ K562 cells. Cells were treated GSH and GSSG assay kit, then detected at 412 nm on the SpectraMax M5/M5e. Error bars represent s.d. from at least three independent experiments. *: *P* < 0.05, **: *P* < 0.01, ***: *P* < 0.001.

Mitochondria are a major source for generation of the reactive oxidative molecules [20]. We have recently found that K562 leukemia cells acquire an alternative mitophagy capability that empowers the leukemia cells in buffering mitochondrial stress even when canonical autophagy is defective [2]. Carbonyl cyanide *m*-chlorophenylhydrazone (CCCP) is able to increase the proton permeability across the mitochondrial inner membranes, thus dissipating the transmembrane potential and depolarizing the mitochondria, and ultimately causing mitophagy [21, 22]. Although alternative mitophagy contributes to the removal of accumulated mitochondria, challenging the leukemia cells with CCCP still increased ROS [2], suggesting that mitophagy may induce counteracting mechanisms to compensate for the loss of ROS from the removal of mitochondria in the erythroleukemia cells. Interestingly, CCCP-induced mitophagy was also found to cause an increase in Sirt3 level in K562 leukemia cells (Figure 1A). In the *Atg*7-deleted K562 leukemia cells where canonical autophagy is defective, induction of alternative mitophagy by CCCP again caused an increase in Sirt3 (Figure 1A). The response of Sirt3 to CCCP challenge was in accordance with that of p62, which was previously reported to be implicated in oxidative response [23, 24]. These results suggest that mitochondrial oxidative stress is correlated to the upregulation of the mitochondrial protein deacetylase Sirt3 in the leukemia cells.

To address the above observation, we depleted Sirt3 with lipofectamine transfection of small inhibitory RNAs targeting *Sirt*3 gene in both parental and *Atg*7-deleted K562 cells (Figure 1B). Surprisingly, knockdown of Sirt3 did not significantly alter mitochondrial mass (Figure 1C), nor did induce apoptosis in the leukemia cells (Figure 1D). Unexpectedly again, knockdown of Sirt3 caused a significant reduction in ROS levels in both parental and *Atg*7-deleted K562 cells that are defective in canonical autophagy (Figure 1E), suggesting that Sirt3 may be contributed to the maintenance of ROS level and upregulation of ROS level by Sirt3 is independent on canonical autophagy.

To further examine whether depletion of Sirt3 truly causes increased oxidative stress, we measured the formation of malondialdehyde (MDA), an organic compound of reactive species resulted from lipid peroxidation of polyunsaturated fatty acids [25]. The results on the marker for oxidative stress showed that lipid oxidation level was decreased when Sirt3 was knocked down in both parental and *Atg*7-deleted leukemia cells (Figure 1F). Reduced glutathione (GSH) is a major antioxidant that provides reducing equivalents for the glutathione peroxidase to catalyze reduction of lipid hydroperoxides, during which the formation of a disulfide bond between two GSH molecules gives rise to oxidized glutathione (GSSG). When cells are exposed to increased levels of oxidative stress, the ratio of GSH to GSSG will decrease. Therefore, the GSH/GSSG ratio is a useful indicator of cellular oxidative stress. Consistent with the above results, knockdown of Sirt3 significantly increased the ratio of the GSH to the GSSG in the parental K562 cells (Figure 1G). Intriguingly, the level of GSH/GSSG was decreased in *Atg*7^−/−^ K562 cells, which was opposite to that in the parental K562 cells. This suggests a different mechanism in the regulation of oxidation of GSH when ATG7-dependent autophagy is defective in the leukemia cells. Nevertheless, the above results together are on the contrary to the previous studies in solid tumor cells where knockdown of Sirt3 not only caused an increased ROS but also led to apoptosis [10, 19].

Our data thus support an opposite role of Sirt3 in the regulation of ROS in K562 leukemia cells as compared with solid tumor cells. Notably, loss of ATG7 protein by *Atg*7 deletion caused an increased Sirt3 (Figure 1A, 1B), similar to an increase in P62 protein (Figure 1A), a marker often used for functional autophagy and recently reported in stress response [23, 24]. The increase in Sirt3 level by *Atg*7 deletion suggests that autophagy may directly or indirectly regulate Sirt3 level in the leukemia cells. Sirt3 is primarily located in the mitochondria in the cell [26]. However, CCCP-induced mitophagy did not degrade Sirt3 (Figure 1A). This suggests that Sirt3 protein may be degraded by an non-autophagic mechanism even when the mitochondria are degraded by autophagy, reflecting a complexity in autophagic regulation of mitochondrial proteins.

### Autophagic regulation of Sirt3 level involves cytoplasmic not nuclear mechanisms

**Figure 2:**
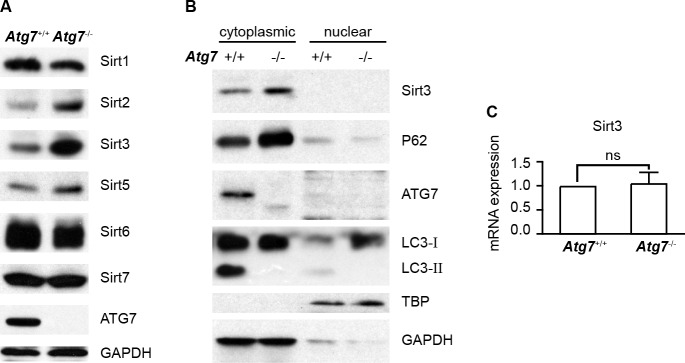
Determination of Sirt3 protein and messenger RNA levels in autophagy-intact or autophagy-defective K562 leukemia cells **A.** Western blotting analysis of Sirtuin family of proteins in *Atg7*^+/+^ and *Atg7*^−/−^ K562 cells. GAPDH served as a protein loading control **B.** Determination of subcellular localization of Sirt3 protein, along with autophagy marker proteins. Cytoplasmic and nuclear proteins were extracted with Thermo's NE-PER nuclear and cytoplasmic extraction reagents. TBP or GAPDH served as a protein loading control for nuclear or cytoplasmic proteins. **C.** Analysis of Sirt3 mRNA level by quantitative real-time polymerase chain reaction. Data shown are representative results from at least three independent experiments. ns: *P* > 0.05.

In order to explore whether autophagy selectively regulates Sirtuin protein family members in K562 leukemia cells, we measured the protein levels of Sirtuin family members in the parental and autophagy-defective K562 leukemia cells. Examination of the proteins by western blotting analysis showed that among six members of the Sirtuin family detected, Sirt3 is one of the members that were most significantly altered in protein levels, with an upregulation in the *Atg*7-deleted cells as compared with their parental cells (Figure 2A), suggesting that autophagic regulation of Sirt3 is largely selective. Western blotting analysis further showed that Sirt3 was restricted to the cytoplasm and *Atg*7 deletion caused an elevated Sirt3 in the cytoplasm; there was no detectable Sirt3 in the nucleus in both the parental and *Atg*7-deleted cells (Figure 2B). Analysis of transcription of *Sirt*3 gene showed that *Atg*7 deletion did not alter *Sirt*3 messenger level (Figure 2C). These data together suggest that Sirt3 level is not regulated by autophagy at transcriptional level, but at posttranslational level presumably in the cytoplasm.

### Activation of autophagy does not directly degrade or downregulate Sirt3

**Figure 3:**
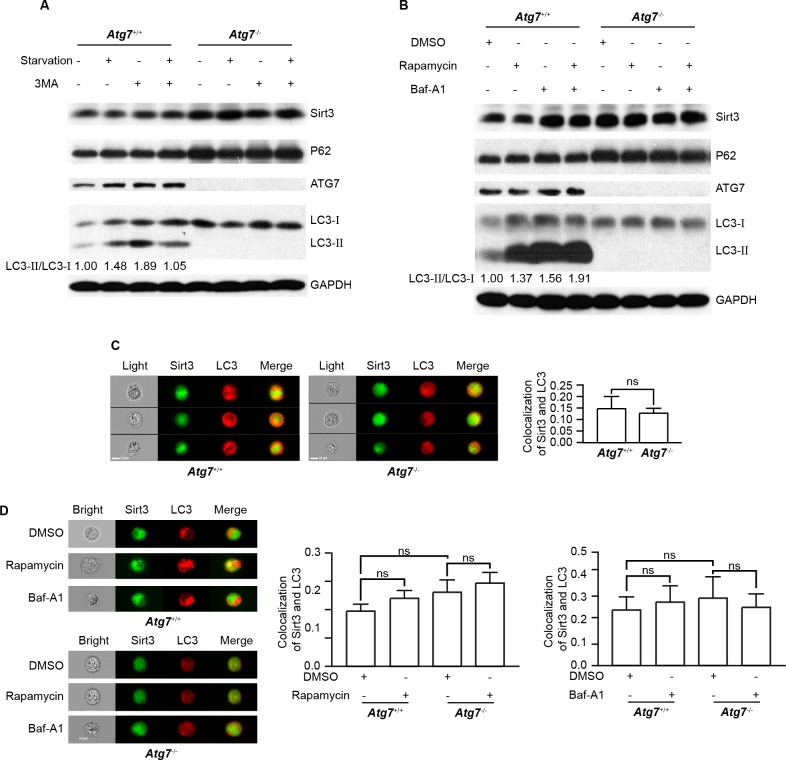
Autophagy does not degrade Sirt3 **A.** Immunoblotting analysis of Sirt3 and autophagy marker proteins in response to starvation and 3-MA in *Atg7*^+/+^ and *Atg7*^−/−^ K562 cells. Cells were starved with HBSS or 500 μM 3-MA alone or in combination for 3 h. GAPDH served as a protein loading control. Quantified ratio of LC3-II/LC3-I was indicated below the blot. **B.** Immunoblotting analysis of Sirt3 and autophagy marker proteins in response to rapamycin or/and bafilomycin A1 in *Atg7*^+/+^ and Atg7^−/−^ K562 cells. Cells were treated with 200 ng/ml rapamycin 3 h or 10 nM bafilomycin A1 alone or in combination overnight. **C.** Image Flow cytometric analysis of colocalization of Sirt3 and LC3 in *Atg7*^+/+^ and *Atg7*^−/−^ K562 cells. After fixation and permeabilization, cells were stained with primary antibody for 30 min, then stained with DyLight 488 conjugated of Goat anti-rabbit IgG(H+L) or DyLight 649 conjugated of Goat anti-mouse IgG(H+L) for 30 min, and finally explored to Amnis ImageStreamX Mark II for image flow cytometry. Samples were visualized and analyzed for the expression of marker proteins with Amnis IDEAS 6.0 software. Left panel: representative flow images, right panel: statistic results. **D.** Image flow cytometric analysis of colocalization of Sirt3 and LC3 in *Atg7*^+/+^ and *Atg7*^−/−^ K562 cells treated with rapamycin or bafilomycin A1. Left panel: representative flow images, middle panel: statistic results from rapamycin treatment, right panel: statistic results from bafilomycin A1 treatment. Data shown are results from at least three independent experiments. ns: *P* > 0.05.

Ubiquitination-proteasomal pathway and autophagy are two major cellular mechanisms for protein degradation. Sirt3 maintains a relative low basal level in K562 leukemia cells. The upregulation of Sirt3 upon *Atg*7 deletion motivated us to examine whether autophagy limits the low basal Sirt3 level by constitutive autophagic degradation of the protein. Surprisingly, while loss of autophagy by *Atg*7 deletion increased Sirt3 level, starvation, a common autophagy stimulus, did not cause Sirt3 reduction, nor did treatment with 3-methyladenine (3-MA), an autophagy inhibitor, cause an accumulation of Sirt3 in the parental K562 leukemia cells (Figure 3A). Similarly, starvation or 3-MA did not alter Sirt3 level in *Atg*7-deleted K562 leukemia cells. Again, P62 displayed a similar pattern to Sirt3 in response to various autophagy stimuli (Figure 3A). Induction of autophagic response was apparent in this experiment scenario since starvation caused an enhanced LC3-II/LC3-I conversion (Figure 3A). In addition, 3-MA promoted LC3-II/LC3-I conversion in non-starvation condition but inhibited the starvation-induced conversion LC3-II/LC3-I (Figure 3A), which was in agreement with the dual role of 3-MA previously reported [27]. These data suggest that starvation-induced autophagy does not directly degrade Sirt3 protein.

To further support the above observation, we treated the parental and *Atg*7-deleted K562 cells with rapamycin, an autophagy inducer that inhibits the kinase mammalian target of rapamycin. Consistent with the starvation data, rapamycin did not reduce Sirt3 level, further suggesting that autophagy does not directly degrade Sirt3 protein (Figure 3B). Both results from starvation and rapamycin treatment support that a non-autophagic mechanism may be involved in the direct degradation of Sirt3 protein.

Surprisingly, treatment with bafilomycin A1, an autophagy inhibitor that inhibits the fusion between autophagosome and lysosome, caused an elevated Sirt3 level in the non-starved leukemia cells. The accumulation of Sirt3 by bafilomycin A1 was associated with autophagic flux blockade in the leukemia cells treated with or without rapamycin (Figure 3B). This suggests that bafilomycin A1 was able to effectively inhibit basal autophagy in non-rapamycin-treated cells and also inhibit induced autophagy in rapamycin-treated cells. Since blockade of autophagy activity by bafilomycin A1 inhibited the degradation of Sirt3, the presumable non-autophagic Sirt3 degradation mechanism appears to require autophagic activity, in particular basal autophagy activity.

Examination of where a protein of interest is recruited to autophagosome assembly site is also a supportive way to answer if the protein of interest is degraded by autophagy. To this end, image flow cytometric analysis was conducted, and the result showed that in the parental K562 cells, the colocalization between Sirt3 and autophagic marker LC3 was hardly detectable and *Atg*7 deletion did not alter this pattern (Figure 3C). The statistical data from image flow cytometric analysis suggested that basal autophagy did not degrade Sirt3, supporting a non-autophagic degradation of Sirt3. Consistent with the western blotting results from the leukemia cells either starved or rapamycin-treated, image flow cytometric analysis also showed that induction of autophagy with rapamycin, or inhibition on autophagy with bafilomycin A1 did not alter the colocalization between Sirt3 and LC3 in both the parental and *Atg*7^−/−^ K562 cells (Figure 3D), further supporting non-autophagic degradation of Sirt3. Therefore, neither basal nor induced autophagy directly degrades Sirt3; however, an unidentified mechanism for non-autophagic degradation of Sirt3 depends on ATG7-dependent basal autophagy activity.

### Basal autophagy limits ubiquitination-proteasomal degradation of Sirt3

**Figure 4:**
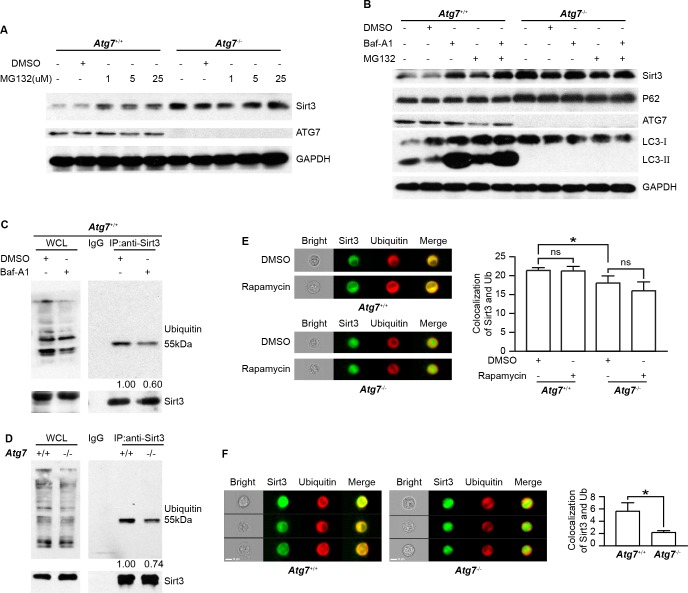
ATG7-dependent autophagy limits ubiquitination-proteasomal degradation of Sirt3 **A.** Immunoblotting analysis of Sirt3 and ATG7 proteins in MG132-treated *Atg7*^+/+^ and *Atg7*^−/−^ K562 cells. Cells were treated with MG132 of indicated concentrations for 6 h. Shown are the representative blots. GAPDH served as a protein loading control. **B.** Immunoblotting analysis of Sirt3 and autophagy marker proteins in *Atg7*^+/+^ and Atg7^−/−^ K562 cells treated with bafilomycin A1 or MG132 alone or in combination. Cells were treated with 5 μM MG132 for 3 h or 10 nM bafilomycin A1 alone or in combination overnight. Shown are the representative blots. GAPDH served as a protein loading control. **C.** Co-immunoprecipitation of Sirt3 with ubiquitin in *Atg7*^+/+^ cells treated with or without bafilomycin A1. Cells were treated with carrier DMSO or 10 nM bafilomycin A1. Cell lysates were prepared using cell lysis buffer for western blotting and Co-immunoprecipitation assay. Proteins were incubated with Sirt3 antibody overnight at 4°C, then incubated with protein A+G agarose for 2 h at 4°C. The purified proteins were detected with western blotting. Left panel: western blotting results of the input for whole cell lysate used as positive control; right panel: Co-IP between Sirt3 and ubiquitin, with relative level of quantification indicated below. IgG used as a negative control. The lower panels are western blotting results of Sirt3. **D.** Co-immunoprecipitation of Sirt3 with ubiquitin in *Atg7*^+/+^ and *Atg7*^−/−^ K562 cells. Left panel: western blotting results of the input for whole cell lysate used as positive control; right panel: Co-immunoprecipitation between Sirt3 and ubiquitin, with relative level of quantification indicated below. IgG used as a negative control. The lower panels are western blotting results of Sirt3. **E.** Image Flow cytometric analysis of colocalization of Sirt3 and ubiquitin in *Atg7*^+/+^ K562 cells treated with carrier DMSO or rapamycin. After fixation and permeabilization, cells were stained with primary antibody against Sirt3 and ubiquitin for 30 min, then stained with DyLight 488 conjugated of Goat anti-rabbit IgG(H+L) or DyLight 649 conjugated of Goat anti-mouse IgG(H+L) for 30 min, then cells were subject to Amnis image flow cytometry. Samples were visualized and analyzed for the expression of marker proteins with Amnis IDEAS 6.0 software. **F.** Image Flow cytometric analysis of colocalization of Sirt3 and ubiquitin in *Atg7*^+/+^ and *Atg7*^−/−^ K562 cells. Data shown are representative results from at least three independent experiments. ns: *P* > 0.05, *: *P* < 0.05.

To test whether Sirt3 is degraded by ubiquitin-proteasomal pathway, we treated the parental and *Atg*7^−/−^ K562 cells with MG132, an inhibitor for proteasomal activity. The result showed that Sirt3 accumulated in the parental K562 cells upon MG132 treatment (Figure 4A), suggesting that proteasomal pathway is responsible for the degradation of Sirt3. Loss of autophagy due to *Atg*7 deletion led to an elevated Sirt3; however, MG132 treatment could not further accumulate Sirt3 in the *Atg*7^−/−^ cells (Figure 4A). This data indicates that once basal autophagy is defective due to *Atg*7 deletion, there was no longer proteasomal degradation of Sirt3, suggesting that the proteasomal degradation of Sirt3 depends on an intact autophagy machinery. In agreement with the above observation, treatment with bafilomycin A1 accumulated Sirt3 (Figures 3B, 4B); however, MG132 was hardly able to enhance the bafilomycin A1-caused accumulation of Sirt3 (Figure 4B). In *Atg*7-deleted K562 cells, neither bafilomycin A1 nor MG132 was able to further accumulate Sirt3 (Figure 4B). This again suggests that the putative proteasomal degradation of Sirt3 requires basal autophagy activity. In contrast, p62 level did not reveal detectable change in response to these pharmacological treatments (Figure 4B). These data also suggest that the putative proteasomal degradation of Sirt3 is selective.

If Sirt3 is indeed degraded by ubiquitination-proteasomal pathway, one would expect a possible reduction in ubiquitination of Sirt3 when the leukemia cells are treated with bafilomycin A1 that accumulated Sirt3. To address this question, we performed co-immunoprecipitation assay between Sirt3 and ubiquitin with K562 leukemia cells treated with or without bafilomycin A1. The result shows that bafilomycin A1 reduced the ubiquitin binding to Sirt3 (Figure 4C), presumably an essential step for proteasomal degradation of this protein. Co-immunoprecipitation assay further show that in the *Atg*7^−/−^ leukemia cells, the amount of ubiquitin binding to Sirt3 was significantly decreased as compared with that in the parental cells (Figure 4D), whereas image flow cytometric analysis show that rapamycin treatment did not have an effect on the colocalization between Sirt3 and ubiquitin (Figure 4E). These results suggest that loss of basal autophagy due to *Atg*7 deletion reduces ubiquitination of Sirt3. Furthermore, we used quantitative image flow cytometry to analyze the effect of basal autophagy activity on the ubiquitination of Sirt3. The representative image and statistic data show that *Atg*7 deletion caused a significant reduction in the localization between Sirt3 and ubiquitin (Figure 4F), further suggesting that basal autophagy defect cause reduced ubiquitination-proteasomal degradation of Sirt3 in the leukemia cells.

The above data thus propose that Sirt3 is directly degraded by ubiquitination-proteasomal pathway but not by autophagic degradation. Loss of autophagy rendered the ubiquitination of Sirt3, reducing proteasomal degradation of Sirt3. Therefore, autophagy, in particular basal autophagy, confers ubiquitination-proteasomal activity to limit Sirt3 level in K562 leukemia cells.

Oxidative lesions are constantly formed in all living cells. DNA, proteins, lipids and kinase signaling pathways are the well-known cellular targets of ROS. Any imbalance between ROS production and the detoxification of their reactive intermediates causes oxidative stress. Cells must respond to this imbalance before the highly reactive molecules damage cellular structures. Sirt3 is a major mitochondrial deacetylase [28], regulating mitochondrial metabolism and ROS [7, 8, 29]. Human Sirt3 is expressed as a full-length 44-kD protein that is targeted to the mitochondria by its N-terminal localization sequence [26, 30]. In mitochondria, Sirt3 is cleaved *via* the mitochondrial matrix processing peptidase to a short 28-kD protein, which is important for Sirt3 enzymatic activity [26, 31, 32]. Recent study has reported that only full-length but not short form of Sirt3 was degraded by ubiquitin-proteasome system (UPS) pathway [33].

In our present study, only a short form of Sirt3 is detectable and subject to autophagy-UPS regulation in K562 leukemia cells. We have recently identified that erythroleukemia cells are able to execute an alternative mitophagy to counteract cellular stress regardless of their conventional autophagy being functional or impaired [2]. Contrary to what has been repeatedly reported in solid tumor cells, we find that Sirt3 functions negatively in relieving oxidative stress and K562 leukemia cells are also able to limit ROS level by autophagy-dependent proteasomal degradation of Sirt3, suggesting that K562 leukemia cells possess multiple mechanisms pertinent to autophagy in buffering cellular stresses, reflecting a leukemic advantage in autophagy. This finding amends our understanding in the unique biology of the leukemia cells in limiting oxidative stress, and hopefully provides a rationale for future targeted therapy on certain type of erythroleukemia.

## MATERIALS AND METHODS

### Cell lines and culture conditions

K562 cell line obtained from ATCC (Manassas, VA, USA) were grown in RPMI-1640 medium (Hyclone, GE healthcare, South Logan, Utah, USA) with 10% fetal bovine serum (Gibco, Thermo fisher scientific, Waltham, MA, USA) in 37°C, 5% CO2 incubator.

### siRNA transfection

Sirt3 was knocked down in *Atg7*^+/+^ and *Atg7*^−/−^ cells by small inhibitory RNA (siRNA, Genepharma, Shanghai, China). Cells were transfected with lipofectamine RNAiMAX (Invitrogen, Thermo fisher scientific, Waltham, MA, USA) according to the manufacturer's instruction. The effects of siRNA on the expression of Sirt3 were evaluated by western blotting at 72 h after transfection. The sequences of the siRNAs: CCAGUGGCAUUCCAGACUUTT/CCAGUGGCAUUCCAGACUUT; GCCCGACAUUGUGUUCUUUTT/AAAGAACACAAUGUCGGGCTT; AAAGAACACAAUGUCGGGCTT/UAAAUGUAUUUCAUGCUGGTT.

### Western blotting analysis

Protein was resolved by 12% SDS-PAGE and transferred to PVDF membranes. The membranes were blocked with 5% skim milk-TBS-0.1% Tween 20 for 1 h at room temperature. Antibodies against Sirt3, TBP, Sirtuin Antibody Sampler Kit (Cell Signaling Technology, Danvers, MA, USA), ATG7 (Abcam, Cambridge, MA, USA), LC3 (Novus Biologicals, Danvers, CO, USA), Ubiquitin (Santa Cruz Biotechnology, Paso Robles, CA, USA), P62 (MBL, Woburn, MA, USA) and GAPDH (Proteintech, Rosemont, IL, USA) were applied to probe the membranes, respectively. The membranes were then washed five times in TBST and incubated with HRP-conjugated secondary antibodies (anti-mouse or anti-rabbit, Cell Signal Technology, USA) diluted 1:2,000 in TBST for 1 h. After 5 times washes, the membranes were developed using an ECL kit (Biological Industries, Kibbutz Beit-Haemek, Israel).

### Co-Immunoprecipitation

Co-Immunoprecipitation (Co-IP) of Sirt3 with ubiquitin in *Atg7*^+/+^ and *Atg7*^−/−^ K562 cells. Cells were splited using cell lysis buffer for Western and IP without inhibitors (Beyotime, Nantong, China). Proteins were incubated with Sirt3 antibody (Cell Signaling Technology, Danvers, MA, USA) overnight at 4°C, then incubated with Protein A+G Agarose (Beyotime, Nantong, China) for 2 h at 4°C. The purified proteins were detected with western blotting. Left panel: western blotting results of the input for whole cell lysate used as a positive control; right panel: Co-IP between Sirt3 and ubiquitin. IgG (Cell Signaling Technology, Danvers, MA, USA) used as a negative control. The lower panels are western blotting results of Sirt3.

### Flow cytometry

After indicated treatment, cells were stained with 100 nM Mitotracker Deep Red (Invitrogen, Thermo fisher scientific, Waltham, MA, USA) in RPMI 1640 for 30 min at 37°C, then the mean fluorescence intensity (MFI) was detected by flow cytometry. Flow cytometric analysis of apoptosis of the Sirt3-depleted *Atg7*^+/+^ K562 cells. Cells were stained using FITC Annexin V Apoptosis Detection Kit I (BD pharmingen, Franklin Lakes, New Jersey, USA) according to the manufacturer's instruction. Flow cytometric analysis of ROS level in the Sirt3-depleted *Atg7*^+/+^ and *Atg7*^−/−^ K562 cells. Cells were stained at 10 μM DCF-DA (Sigma-Aldrich, St. Louis, MO, USA) for 15 min at 37°C.

### Imagestream analysis

Image flow cytometric analysis of colocalization of Sirt3 and ubiquitin. After fixation and permeabilization using 0.1% triton-100X (BDGCS biotechnology, Beijing, China), cells were stained with primary antibody against Sirt3 (Cell Signaling Technology, Danvers, MA, USA) and ubiquitin (Abcam, Cambridge, MA, USA) for 30 min, then stained with DyLight 488 conjugated of Goat anti-rabbit IgG(H+L) or DyLight 649 conjugated of Goat anti-mouse IgG(H+L) (Multi Sciences, ZheJiang, China) for 30 min, then cells were explored to ImageStreamX Mark II (Amnis, Merck Millipore, Seattle, WA, USA) for image flow cytometry. Samples were visualized and analyzed for the expression of marker proteins with IDEAS 6.0 software (Amnis, Merck Millipore, Seattle, WA, USA).

### MDA assay

Lipid perxodisation was determined by MDA assay in the Sirt3-depleted *Atg7*^+/+^ and *Atg7*^−/−^ K562 cells. Cells were treated according to the Lipid Peroxidation MDA Assay Kit (Beyotime, Nantong, China) instruction, then detected at 532 nm on SpectraMax M5/M5e (Molecular devices, Sunnyvale, CA, USA).

### GSH/GSSG assay

Analysis on the ratio of GSH to GSSG in the Sirt3-depleted *Atg7*^+/+^ and *Atg7*^−/−^ K562 cells. Cells were treated according to the GSH and GSSG Assay Kit (Beyotime, Nantong, China) instruction, then detected at 412 nm on SpectraMax M5/M5e (Molecular devices, Sunnyvale, CA, USA).

### mRNA analysis

Analysis of Sirt3 mRNA level by quantitative real-time polymerase chain reaction. Primers Sirt3-forward CATTCGGGCTGACGTGATG, Sirt3-reverse AACCACATGCAGCAAGAACCT. Data shown are representative results from at least three independent experiments. Two-tailed student's t-test was used to determine statistical significance (ns, P > 0.05).

### Reagents

Cells were incubated with 20 μM CCCP (Sigma-Aldrich, St. Louis, MO, USA), 10 nM bafilomycin A1 (Sigma-Aldrich, St. Louis, MO, USA), MG-132 (Merck Millipore, Seattle, WA, USA), 500 μM 3-MA (Sigma-Aldrich, St. Louis, MO, USA), 200 ng/ml rapamycin (Selleckchem, Houston, TX, USA), HBSS (Gibco, Thermo fisher scientific, Waltham, MA, USA),

### Statistical analysis

The data are presented as mean values from three separate experiments ± s.d. Statistical analysis are performed using GraphPad Prism 5 Software. Error bars represent SEM and p values calculated with a two-tailed Mann-Whitney test unless stated otherwise. (ns, no significance, *P < 0.05, **P < 0.01, ***P < 0.001).
